# Supplementary material for: Steady-State Levels of Cytokinins and Their Derivatives May Serve as a Unique Classifier of Arabidopsis Ecotypes
Source: Plants (Basel). 2020 Jan 17;9(1):116. doi: 10.3390/plants9010116 (PMC7020191; doi:10.3390/plants9010116)
Supplement: Supplementary file 1 [file plants-09-00116-s001.pdf]

Table S1: Calculated non – significant correlations between hormonal levels and environmental factors at site of origin of the ecotype.

		Correlation coefficient	p-value
Shoots	CKs – Altitude	0.12	0.53
	IAA – Altitude	0.07	0.71
	ABA/CKs - Altitude	-0.03	0.88
	ABA – Precipitation	-0.29	0.11
	CKs – Precipitation	-0.13	0.48
	IAA – Precipitation	-0.23	0.22
	ABA/CKs – Precipitation	-0.28	0.12
	CKs – Temperature	-0.045	0.81
	IAA – Temperature	0.19	0.31
Roots	CKs – Altitude	0.21	0.25
	IAA – Altitude	-0.09	0.61
	ABA/CKs - Altitude	0.14	0.46
	ABA – Precipitation	-0.12	0.49
	CKs – Precipitation	-0.15	0.43
	IAA – Precipitation	-0.006	0.97
	ABA/CKs – Precipitation	-0.05	0.78
	CKs – Temperature	0.136	0.47
	IAA – Temperature	-0.063	0.74
	ABA/CKs – Temperature	-0.134	0.48
	ABA – Temperature	-0.026	0.89

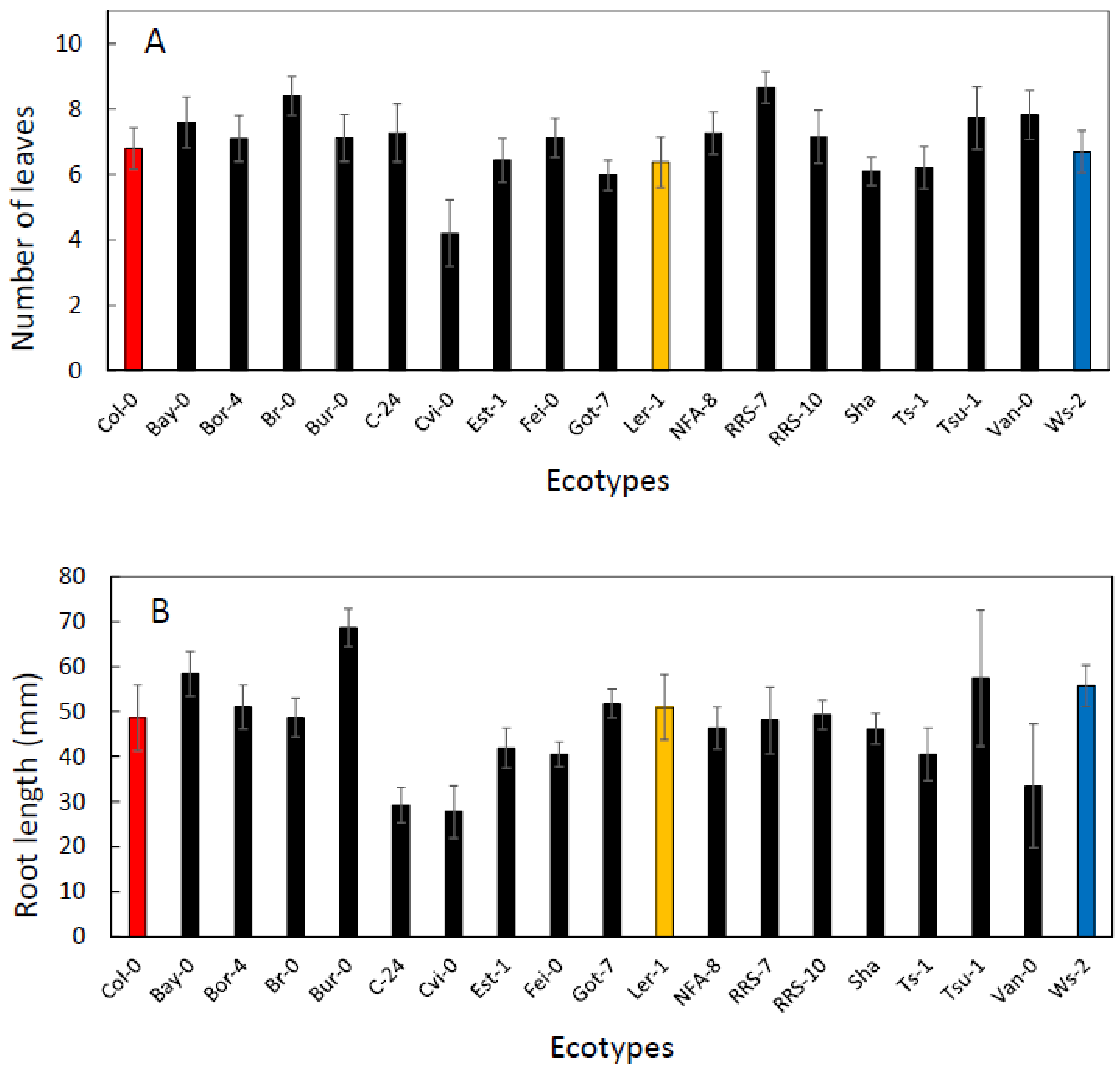

Figure S1. The number of true leaves at 21 days after sowing (A) and the length of primary root 14 DAS (B) among selected *A. thaliana* ecotypes grown in controlled conditions in preliminary experiment. Commonly used ecotypes are marked in colour (Red = Col-0, Blue - Ws-0, Orange – Ler-1). See Table 1 for the full list of ecotypes. Means and SD are shown.

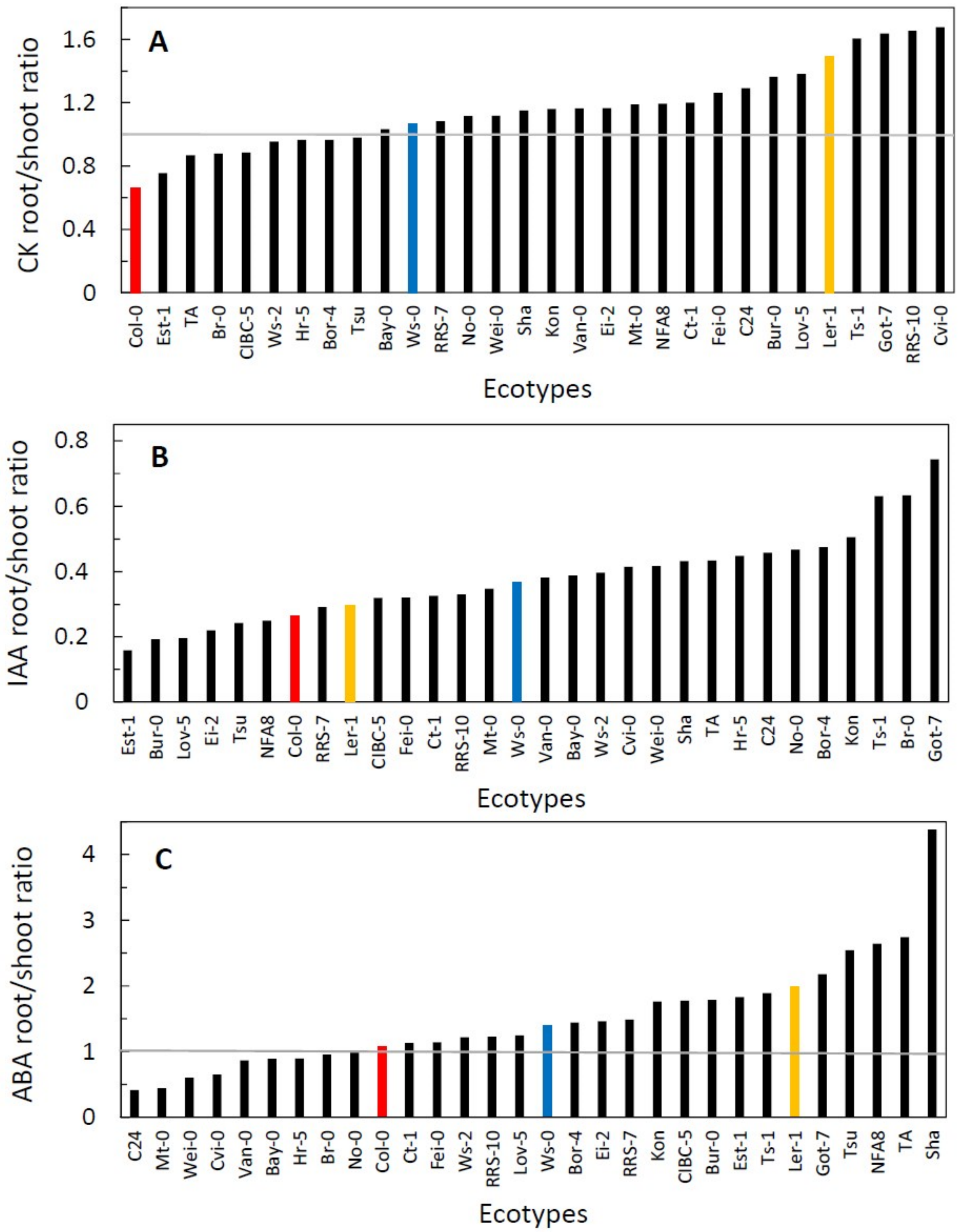

Figure S2. **The ratio of contents of CKs (A), IAA (B) and ABA (C) in root and shoot** after 21d cultivation in controlled conditions. The ecotypes of *A. thaliana* plants were ranked from the lowest to the highest ratio. Commonly used ecotypes are marked in colour (Red = Col-0, Blue - Ws-0, Orange – Ler-1). See Table 1 for the full list of ecotypes.

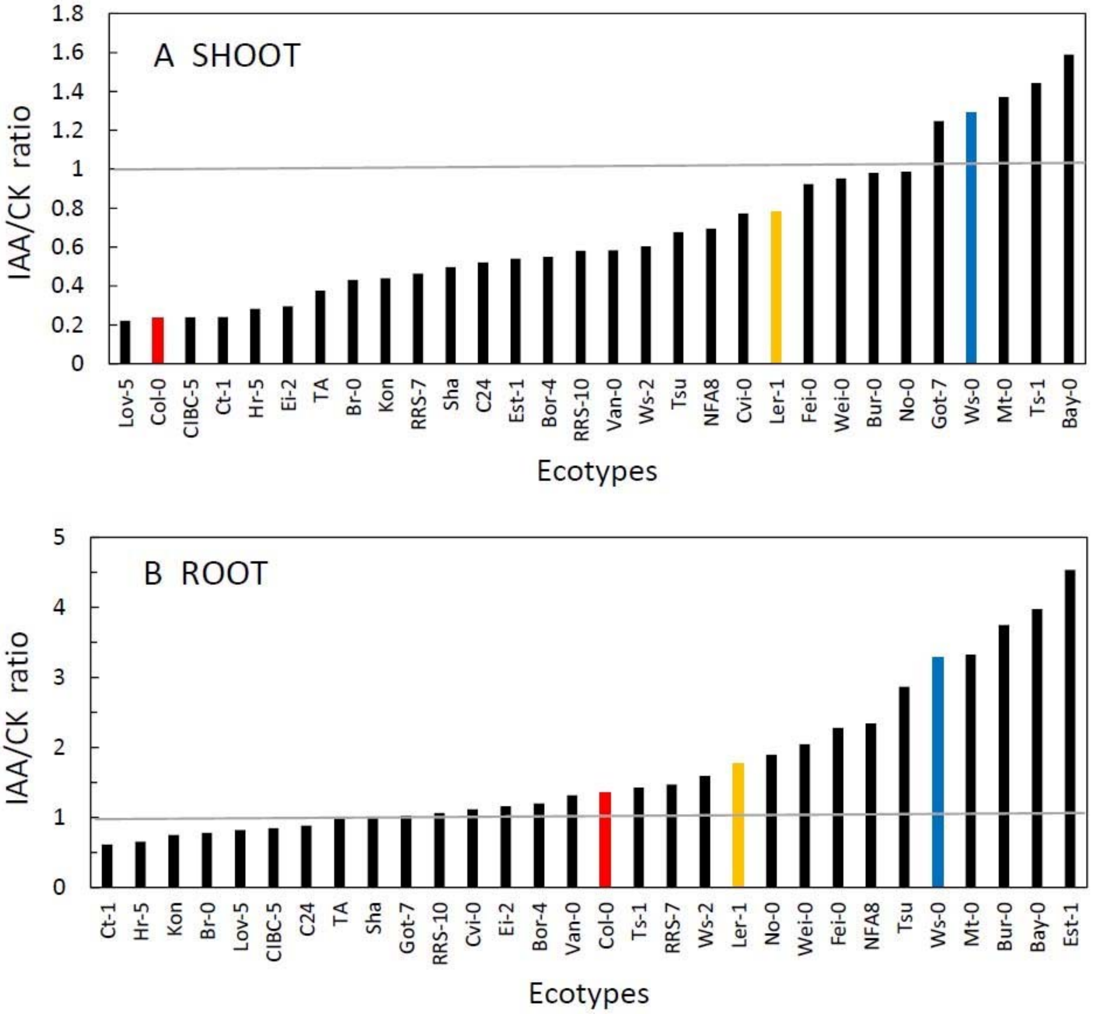

Figure S3. The ratio of IAA and total CKs in the shoot (A) and the root (B) ranked from the lowest to the highest among thirty *A. thaliana* ecotypes grown 21d in controlled conditions. Commonly used ecotypes are marked in colour (Red = Col-0, Blue - Ws-0, Orange – Ler-1). See Table 1 for the full list of ecotypes.
